# New network topology approaches reveal differential correlation patterns in breast cancer

**DOI:** 10.1186/1752-0509-7-78

**Published:** 2013-08-15

**Authors:** Michael Bockmayr, Frederick Klauschen, Balazs Györffy, Carsten Denkert, Jan Budczies

**Affiliations:** 1 Institute for Pathology, Charité University Hospital Berlin, Charitéplatz 1, 10117 Berlin, Germany; 2 Joint Research Laboratory of the Hungarian Academy of Sciences and the Semmelweis University, Semmelweis University 1st Dept of Pediatrics, 1083 Budapest, Hungary

**Keywords:** Differential correlation, Microarray data, Breast cancer, Molecular subtypes, Differential co-expression

## Abstract

**Background:**

Analysis of genome-wide data is often carried out using standard methods such as differential expression analysis, clustering analysis and heatmaps. Beyond that, differential correlation analysis was suggested to identify changes in the correlation patterns between disease states. The detection of differential correlation is a demanding task, as the number of entries in the gene-by-gene correlation matrix is large. Currently, there is no gold standard for the detection of differential correlation and statistical validation.

**Results:**

We developed two untargeted algorithms (DCloc and DCglob) that identify differential correlation patterns by comparing the local or global topology of correlation networks. Construction of networks from correlation structures requires fixing of a correlation threshold. Instead of a single cutoff, the algorithms systematically investigate a series of correlation thresholds and permit to detect different kinds of correlation changes at the same level of significance: strong changes of a few genes and moderate changes of many genes. Comparing the correlation structure of 208 ER- breast carcinomas and 208 ER+ breast carcinomas, DCloc detected 770 differentially correlated genes with a FDR of 12.8%, while DCglob detected 630 differentially correlated genes with a FDR of 12.1%. In two-fold cross-validation, the reproducibility of the list of the top 5% differentially correlated genes in 140 ER- tumors and in 140 ER+ tumors was 49% for DCloc and 33% for DCglob.

**Conclusions:**

We developed two correlation network topology based algorithms for the detection of differential correlations in different disease states. Clusters of differentially correlated genes could be interpreted biologically and included the marker genes hydroxyprostaglandin dehydrogenase (PGDH) and acyl-CoA synthetase medium chain 1 (ACSM1) of invasive apocrine carcinomas that were differentially correlated, but not differentially expressed. Using random subsampling and cross-validation, DCloc and DCglob were shown to identify specific and reproducible lists of differentially correlated genes.

## Background

Over the last 15 years, global gene expression profiling using microarrays has been established as a common tool for disease research. With this approach, disease mechanisms may be studied by comparative expression profiling of disease and healthy tissues or two disease states A and B. In recent years, this approach helped to discover prognostic markers and signatures and to identify target structures for drug intervention. Alterations of gene regulation often result in up- or down-regulated genes. Therefore, looking for differentially expressed genes using statistical tests is one of the most common strategies for the comparative analysis of microarray data [1].

However, this approach ignores the fact that most of the biological processes require orchestrated action of many genes. Therefore, gene correlation and co-expression have been intensively studied since the early days of microarrays and the seminal work of Eisen et al. [2]. Today, hierarchical clustering and heatmaps are ubiquitous in studies of microarray data. Heatmaps usually serve as a tool for visualization of the results. Clustering has also been shown to be useful for the identification of disease subtypes, such as, for instance, defining molecular subtypes of breast cancer [3].

Complementary to differential expression analysis, the study of differential correlation or differential co-expression aims at a deeper understanding of the expression patterns in diseased tissues. As an example, a number of downstream targets could be regulated by a master gene, for example a transcription factor. In tissues where the regulatory mechanism is functional, the module of the target genes will be expressed in an ordered pattern. However, in diseased tissues where the regulatory mechanism is dysfunctional, the expression of the gene module will be unordered or random. Correlation changes of this kind can be detected by differential correlation (DC) analysis, but might be overlooked by differential expression (DE) analysis.

The number of pairwise correlations in global expression data of human tissues is quadratic in the number of genes and exceeds one million. Case-by-case testing would lead to a multiple testing problem, connected with searching for a few differentially correlated gene pairs within a huge number of unregulated correlations. Therefore, it should be more efficient not to study each gene pair separately, but to take into account the overall structure of correlations. Shortly after the microarray technology became common, algorithms for the detection of differential co-expression and differential correlation were developed [4-6]. Meanwhile, a multitude of algorithms were published [7] that can be divided into targeted, semi-targeted and untargeted approaches [8].

In targeted approaches, predefined gene modules are analyzed for correlation changes between the two disease states. Frequent choices for the modules are Gene Ontology (GO) categories, Kyoto Encyclopedia of Genes and Genomes (KEGG) pathways, or clusters from additional external expression data sets. For example, in gene set co-expression analysis (GSCA) a dispersion index is calculated for each of the modules and the significance is assessed using a permutation test [9]. In [10], a difference network framework is developed and a test statistics is defined by averaging over the edge weights between members of the modules. In another kind of targeted approach, the analysis of correlations is restricted to a predefined network, for example to the human interactome [11]. In [12], the expression pattern of breast cancer on the interactome network was analyzed and it was shown that the metastatic cancer phenotype is characterized by an increase of randomness of the local information flux patterns.

In semi-targeted approaches, modules in one of the disease states are defined using clustering, and these modules are investigated for correlation in the other disease state. The differential clustering algorithm (DCA) starts with clustering of the tissues in the reference disease state and proceeds with reordering the genes in the reference clusters according to the correlations in the second disease state [13]. CoXpress starts with hierarchical clustering of the reference samples and proceeds with a resampling-based approach to find those modules that are co-expressed in one state, but not in the other [14].

Untargeted approaches do not depend on externally defined modules or modules defined by clustering of a reference data set. Therefore, untargeted algorithms are capable of detecting DC in more general situations where differential regulation neither occurs within predefined external nor internal modules. Many of the untargeted approaches start with constructing correlation (or interaction) networks of each of the disease states and proceed with identification of differentially correlated subnetworks [15-18]. The recently published DICER algorithm [19] is able to address two different scenarios of DC: differentially correlated clusters, but also differentially correlated meta-modules. Here, a meta-module is defined as a pair of gene sets with genes inside the sets being correlated in both disease states, but with differing correlations between the gene sets.

Transformation of a correlation structure into a network requires fixing of a threshold. Whenever a correlation exceeds the threshold, the corresponding two network nodes are joined. A novelty of the current study is to investigate changes in network topology, but at the same time to evaluate a series of correlation thresholds that comprehensively cover the range of correlations in the data. In this way, different kinds of correlation changes can be detected at the same level of significance: strong changes of a few genes, but also moderate changes of many genes.

**Figure 1:**
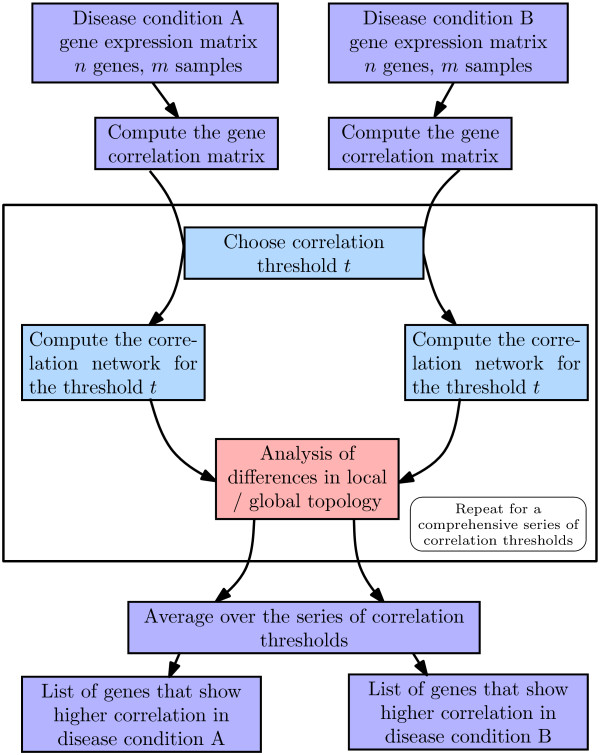
**Workflow of the algorithms for detection of differentially correlated genes.** In the first step, the gene correlation matrix is calculated for each of the disease conditions. In the second step, correlation networks are constructed for a fixed correlation threshold. Two genes are connected with an edge whenever the Pearson correlation exceeds this threshold. The differences in local (algorithm DCloc) or global (algorithm DCglob) topology of the networks are analyzed. Step two is repeated for a series of thresholds (typically 100) such that a good coverage of the correlations in the data set is obtained. The series of thresholds is chosen as equidistant sequence of Fisher-transformed correlations. In the third step, the results for the thresholds are averaged and a measure of differential correlation is calculated for each of the genes. After choosing a cutoff point for the measure of differential correlation, a list of genes with higher correlation in condition A and a list of genes with higher correlation in condition B are obtained.

We designed two untargeted algorithms that numerically quantify the DC of each gene. Each algorithm delivers an ordered gene list according to the strength of DC between the two disease states. The first algorithm aims at the detection of global changes of the network topology (DCglob), the second at the detection of local changes (DCloc). The workflow of the algorithms is shown in Figure 1. In a first step, correlation networks are constructed for disease states A and B. Second, the DC of each of the genes is calculated as global or local topological change between the networks. Third, the analysis is repeated for 100 (or 200) correlation thresholds and the results are averaged. Finally, ranked lists of differentially correlated genes are obtained for both algorithms. False discovery rates (FDR) for the resulting gene lists are estimated using a random subsampling method.

Worldwide, breast cancer is classified into molecular subtypes based on estrogen receptor (ER) and HER2 status. Determination of the molecular subtype is essential to tailor adjuvant treatment and to estimate of the risk of recurrence after surgery. In the last decade, DE between molecular subtypes of breast cancer was extensively investigated [20-22]. However, the literature on DC analysis of breast cancer is limited and includes a de-novo partitioning method [23] and a targeted analysis of KEGG pathways [24]. Therefore, we tested the new developed untargeted algorithms in a large gene expression data set of 208 ER-, 208 ER+, 208 HER2- and 208 HER2+ breast carcinomas.

## Methods

The algorithms DCglob and DCloc (Additional file 1) were implemented using the statistical programming language R[25]. While the global algorithm focuses on comparison of the connected components of the networks, the local algorithm compares the next neighbors of the gene under consideration. The general workflow of the algorithms is illustrated in Figure 1.

### Computing time

Calculations were done on a Linux computer including 16 GB RAM and an Intel Core i7-2600 processor, 3.40 GHz. In the first step, the gene correlation matrix was calculated and used as input for both of the algorithms. Because this calculation did not significantly contribute, the computing time was independent of the number of samples. The time to calculate the strength of differential correlation for 12703 genes was 52 minutes for DCglob (200 thresholds) and 69 minutes for DCloc (100 thresholds). Including FDR calculation by subsampling analysis (100 subsamples), the calculation time was 88 hours for DCglob and 116 hours for DCloc.

### Global topology algorithm

**Step 1** The algorithm compares gene expression data of *n* samples in disease condition *A* with gene expression data of *n* samples in disease condition *B*. First, the correlation matrix *C*^*q*^ comprising the Pearson correlations $c_{\text{ij}}^{q}=\text{cor}({\text{gene}}_{i},\;{\text{gene}}_{j})$ of all pairs of genes is calculated for both disease conditions *q*=*A*,*B*. The Fisher-transformed correlation matrix $z_{\text{ij}}^{q}=\frac{1}{2}\cdot \log\frac{1+\overset{q}{\underset{\text{ij}}{c}}}{1-\overset{q}{\underset{\text{ij}}{c}}}$ is the starting point for all further calculations.

### 

**Step 2** Correlation networks are computed for a comprehensive series of correlation thresholds. In the breast cancer data set, the highest correlation between genes is *c*=0.985 and corresponds to a Fisher-transformed value of *z*=2.5. Therefore, we choose the set of thresholds *T* to be the series of 200 equidistant values between 0 and 2.5. For each threshold *t*∈*T*, we obtain correlation networks $N_{A}^{t}$ and $N_{B}^{t}$ corresponding to the disease conditions *A* and *B*.

### 

**Step 3** The decomposition of the networks $N_{A}^{t}$ and $N_{B}^{t}$ into connected components is computed and all connected components containing 3 or more genes are selected. We restricted to clusters of 3 or more genes, because this is the minimum number where the network topology comes into play. Changes of pairwise correlations could be more effectively studied using a direct approach. Formally, let $\left\{A_{1}^{t},\ldots ,A_{k}^{t}\right\}$ and $\left\{B_{1}^{t},\ldots ,B_{l}^{t}\right\}$ denote the sets of these connected components in $N_{A}^{t}$ and $N_{B}^{t}$ respectively. After these preparations, we remove the genes that are contained in a connected component for both networks $N_{A}^{t}$ and $N_{B}^{t}$ from the set of all genes *G*, yielding ${\tilde{G}}^{t}:=G\setminus \underset{1\leq i\leq k,1\leq j\leq l}{⋃}(A_{i}^{t}\cap B_{j}^{t})$. Then, we build subnetworks ${Ñ}_{A}^{t}$ resp. ${Ñ}_{B}^{t}$ of $N_{A}^{t}$ resp. $N_{B}^{t}$ induced by the genes in ${\tilde{G}}^{t}$ and compute the sets ${Ã}^{t}$ and ${\tilde{B}}^{t}$ of genes that are contained in connected components of ${Ñ}_{A}^{t}$ and ${Ñ}_{B}^{t}$ with cardinality greater or equal 3. Because we removed the genes that are contained in correlation clusters for both disease conditions, the remaining genes that are in correlation clusters for one of the disease conditions are considered as differentially correlated for correlation threshold *t*.

To summarize the information about differential correlation for all thresholds, we define indicator functions $I_{j}^{A},I_{j}^{B}:T\to \{0,1\}$ for every *g*_*j*_∈*G* by

$$I_{j}^{A}(t)=\left\{\;\begin{array}{cc} 1, & \text{if}g_{j}\in {Ã}^{t}\\ 0, & \text{else} \end{array}\right)\;\text{and}\;\;\;I_{j}^{B}(t)=\left\{\;\begin{array}{cc} 1, & \text{if}g_{j}\in {\tilde{B}}^{t}\\ 0, & \text{else} \end{array}\right)\text{.}$$

As an example, $I_{j}^{A}(t)=1$ indicates that gene *j* is member of a connected component in the network $N_{A}^{t}$ but not in the network $N_{B}^{t}$.

### 

**Step 4**Finally, genes that are members of connected components in only one of the networks over a large range of correlation thresholds are selected. To this end, an interval of maximal length [ *a*,*b*] is chosen such that $I_{j}^{q}(t)=1$ for all *t*∈ [ *a*,*b*]∩*T*. Thus, the interval contains as series of threshold values for which a gene is correlated in one of the networks, but not in both networks. The interval length *b*−*a* is converted to a *p*-value using Steiger’s test for the comparison of correlation coefficients [26] and used to measure the strength of differential correlation. A list of differentially correlated genes includes all genes with *p*-values *p*<*S* below a threshold *S*.

## Local topology algorithm

**Step 1** This step is identical to Step 1 performed for DCglob.

**Step 2** We choose the set of thresholds *T* to be the series of 100 equidistant values between 0 and 2.5. Let $N_{A}^{t}$ and $N_{B}^{t}$ denote the correlation networks constructed for each threshold *t*∈*T*. For a given gene *i*, let $V_{A}^{i,t}$ resp. $V_{B}^{i,t}$ be the set of neighbors of this gene in $N_{A}^{t}$ resp. $N_{B}^{t}$. We define the topological dissimilarity between the two networks in the neighborhood of gene *i* as:

$$d_{i}^{t}:=1-\frac{\left|V_{A}^{i,t}\cap V_{B}^{i,t}\right|}{\left|V_{A}^{i,t}\cup V_{B}^{i,t}\right|}.$$

In this definition, the number of common next neighbors in both networks is divided by the total number of next neighbors. To focus on changes that affect correlation clusters of at least 3 genes, we set $d_{i}^{t}:=0$ if $|V_{A}^{i,t}\cup V_{B}^{i,t}|<3$.

**Step 3** Finally, the value of differential correlation for each gene is computed by averaging the topological dissimilarity over the thresholds under consideration,

$$d_{i}:=\frac{1}{\mathrm{\#T}}\cdot \underset{t\in T}{\sum }d_{i}^{t}\text{.}$$

Thus, we obtain a value in [0,1], which represents the strength of differential correlation for each gene. A list of differentially correlated genes includes all genes with *d* >*s* above a threshold *s*.

## Estimation of false discovery rates

Statistical evaluation of the algorithms was performed by a repeated random subsampling analysis. We wanted to falsify the null hypothesis that both patients groups exhibit the same gene correlation structure. Therefore, we randomly subsampled arbitrary breast cancer patients to generate the null distribution. This procedure mixes ER+ and ER- patients (as well as HER2+ and HER2- patients) and therefore is appropriate to assess the significance of differential correlations between the ER+ and ER- subtype (as well as the HER2+ and HER2- subtype). Then, we compared the number of differentially correlated genes between breast cancer subtypes *n*_*AB*_ to the number of differentially correlated genes between randomly sampled sets of breast cancer *n*_0_.

In detail, we estimated the expected number of differentially correlated genes under the null hypothesis from 100 random subsamples. We obtained (mean values with standard errors) *n*_0_=76±6 and 38±4 for DCglob (cutoff *p*=0.1,0.05) and *n*_0_=99±8 and 10±1 for DCloc (cutoff *d*=0.25,0.3). Thus, 100 repetitions were enough for precise estimation of *n*_0_. A confidence interval was estimated from the 5% and the 95% percentile of the distribution of *n*_0_. Finally, for each correlation threshold *t*, we estimated

$$\text{FDR}(t)=\frac{\pi_{0}n_{\text{AB}}(t)}{{\bar{n}}_{0}(t)}\approx \frac{n_{\text{AB}}(t)}{{\bar{n}}_{0}(t)},$$

wherein *π*_0_ denotes the proportion of not differentially correlated genes. This is a standard method for estimating the FDR from a subsampling or permutation analysis, see for example [27]. For breast cancer, the number of differentially correlated genes turned out to be small compared to the total number of genes. Therefore, slightly overestimating the FDR, we used the approximation *π*_0_=1.

## Dataset

We generated a large gene expression data set of breast cancer (1317 samples) by fusion of publicly available microarray data sets. Raw data of GSE1456, GSE2034, GSE4922, GSE6532, GSE7390 and GSE11121 with respectively 159, 286, 327, 578, 198 and 200 samples were downloaded from the Gene Expression Omnibus (GEO) website [28]. All the samples were analyzed using the Affymetrix Human Genome U133A microarray. As remarked in [29] some of the samples were contained in two or more data sets. Thus, we removed 431 samples and ended up with a breast cancer gene expression data set of 1317 unique samples. The raw data were preprocessed using the mas5 protocol as implemented in the R package affy[30] and transformed to log2 scale. All samples consisted of surgical collected fresh-frozen tissue of primary tumors without neoadjuvant treatment.

A large number of genes was represented by more than one microarray probe. In this case, we selected the probe with the highest expression level resulting in a gene expression data set of 12703 unique genes. Immunohistochemistry (IHC) and in situ hybridization (ISH) where necessary are the gold standard for the determination of the ER and HER2 status. However, immunohistochemical data of ER and HER2 protein expression were not available for all samples. Hence, ER and HER2 classification was performed using the expression level of the estrogen receptor 1 gene (probe 205225_at) and the HER2 gene (probe 216836_s_at) from the microarray data (Additional file 2). A high concordance between RNA based determination of ER and HER2 states and the IHC based standard method was demonstrated before [22,31]. A value of 10 was chosen as threshold for the ER status and a value of 12 as threshold for the HER2 status.

## Visualization and functional analysis

Heatmaps were generated using the R function heatmap. Hierarchical clustering was executed using the average linkage method with Pearson correlations as similarity measure. Prior to the analysis, the expression level of each gene was centered to mean 0 and standard deviation 1. Construction and analysis of networks was carried out using the R package igraph[32]. Visualization of the networks was realized using Cytoscape[33]. Gene enrichment analysis was executed using DAVID [34,35] with the genes represented by the microarray as background.

## Results

Two algorithms were developed for the detection of differential correlation in different disease states (Figure 1). The algorithms are based on the detection of either global (DCglob) or local (DCloc) changes in the topology of the correlation network. Both algorithms include the analysis of correlation networks corresponding to a series of correlation thresholds that covers the range of correlations in the data.

### Identification of differentially correlated genes

We investigated the differential correlation in the molecular subtypes of breast cancer. To this end, six microarray data sets were downloaded from GEO [28] and joined into a large gene expression cohort of 1317 tumor samples. We divided the microarray cohort into molecular subtypes by the status of estrogen receptor (ER) and HER2. The prevalence of the molecular subtypes in the gene expression cohort was similar to their prevalence in a large population of Californian women (Table 1). The gene expression cohort included 208 HER2+ samples. To obtain comparable results, we worked with the same number of tumors in each of the molecular subgroups and compared 208 ER+ tumors with 208 ER- tumors and 208 HER2+ with 208 HER2- tumors. These subsamples were randomly drawn.

**Table 1:** Distribution of ER and HER2 status

**Microarray cohort**			**Californian population **[36]
Total	1317	(100%)	100%
ER+	1035	(78.6%)	79.4%
ER-	282	(21.4%)	20.6%
HER2+	208	(15.8%)	21.7%
HER2-	1109	(84.2%)	78.3%
ER+/HER2+	120	(9.1%)	14.6%
ER+/HER2-	915	(69.5%)	64.8%
ER-/HER2+	88	(6.7%)	7.1%
ER-/HER2-	194	(14.7%)	13.5%

Distribution of ER and HER2 status in the microarray cohort and in a large Californian population of 67698 breast cancer patients.

The genes were ranked according to the strength of differential correlation *p* (DCglob) and *d* (DCloc), see Additional files 3 and 4. The statistic *p* can be interpreted as the significance of the range of correlations, where the gene under consideration takes part in a change of global topology. The statistic *d* can be interpreted as topological dissimilarity of the networks in the neighborhood of the gene under consideration. Stronger differential correlation corresponds to lower *p*, but higher *d*.

Lists of differentially correlated genes were generated by choosing thresholds for the two statistics (Table 2). For each of the gene lists, the false discovery rate (FDR) was estimated using a random subsampling method. Using DCglob, 630 differentially correlated genes (FDR = 12.1%) were detected between ER subtypes and 804 (FDR = 9.5%) between HER2 subtypes. Using DCloc, 770 differentially correlated genes (FDR = 12.8%) were detected between ER subtypes and 1027 (FDR = 9.6%) between HER2 subtypes. Lower FDRs can be obtained by using more stringent cutoffs (Table 2).

**Table 2:** **Numbers of detected genes by DCgloband by DCloc**

**Algorithm**	**Threshold**	**ER**		**HER2**	
		**Genes**	**FDR**	**Genes**	**FDR**
DCglob	*p*<0.1	630	12.1%	804	9.5%
	*p*<0.05	420	8.9%	544	6.9%
DCloc	*d*>0.25	770	12.8%	1027	9.6%
	*d*>0.3	185	5.4%	238	4.2%

Number of detected genes after choosing the cutoff points *p* = 0.1, 0.05 (DCglob) and *d* = 0.25, 0.3 (DCloc). FDRs were estimated using a repeated random subsampling method.

### Variation of the cutoff parameters

**Figure 2:**
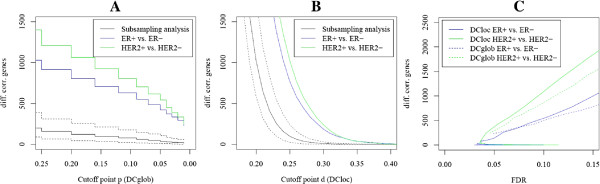
**Dependence of the resulting gene list on the cutoff point.(A)** Algorithm DCglob: Number of detected genes in dependence of the cutoff point for the change of global network topology. The parameter *p* is connected with the length of the interval where the change of correlation is stable. Additionally, the mean number of detected genes comparing 100 pairs of breast cancer random subsamples including a 90% confidence interval is shown. **(B)** Algorithm DCloc: Number of detected genes in dependence of the cutoff point for the changes of local network topology. The parameter *d* can be interpreted as proportion of correlated genes that are not shared by condition A and B. Similarly, the number of detected genes comparing 100 pairs of breast cancer random subsamples including a 90% confidence interval is shown. **(C)** Comparison of DCglob and DCloc: The number of detected genes in dependence of the FDR.

Figure 2 shows the number of differentially correlated genes in dependence of the strength of differential correlation. The number of differentially correlated genes between randomly subsampled groups of breast cancer tissue is shown as baseline. Indeed, there were significantly more differentially correlated genes between molecular subtypes of breast cancer than between random samples of breast cancer. ROC curves show the number of differentially correlated genes in dependence of the FDR (Figure 2C). Over a large range of FDRs, the number of differentially correlated genes between the HER2+ and the HER2- subtype was higher than the number of differentially correlated genes between the ER+ and the ER- subtype. Furthermore, the number of differentially correlated genes detected by DCloc for a fixed FDR value was higher than the number of differentially correlated genes detected by DCglob for both of the subtype comparisons.

### Differential correlation vs. differential expression

We looked for differential expression between the breast cancer subtypes using the standard approach of Welch’s test. After using the Benjamini-Hochberg (BH) method for multiple testing correction and a FDR of 5%, 55% of all genes were differentially expressed between ER+ and ER-, and 31% were significantly differentially expressed between HER2+ and HER2-. Among the differentially correlated genes identified by DCglob (*p*<0.1), 76% were differentially expressed between ER+ and ER- and 48% were differentially expressed between HER2+ and HER2-. For DCloc (*d*>0.25), the percentages were similar (73% and 47%). Thus, DC analysis provided additional information beyond DE analysis. As an example, the marker genes for the invasive apocrine subtype of breast cancer acyl-CoA synthetase medium chain 1 (ACSM1) and hydroxyprostaglandin dehydrogenase (PGDH) exhibit strong differential correlation (DCglob*p*=8.0E-05 and *p*=0.0004; DCloc*d*=0.26 and *d*=0.27), but they would not be detected in a differential expression analysis (*p*=0.21 and *p*=0.55 after Benjamini-Hochberg correction).

### Functional analysis of the differentially correlated genes

We performed a gene enrichment analysis using the bioinformatics tool DAVID [34,35]. We separately submitted the differentially correlated genes between ER+ and ER-, and HER2+ and HER2- (*p*<0.1 for DCglob, *d*>0.25 for DCloc) and identified many overrepresented terms. The most important results are presented in Table 3 for ER, and in Table 4 for HER2. First, there was a significant enrichment in cell cycle genes, which was particularly pronounced in the analysis comparing HER2+ and HER2- breast cancer (*p*<6.8E-13). Genes related to the immune response were also enriched. Next, genes associated with the extracellular matrix (*p*<9.6E-13) were enriched in the differentially correlated genes between ER+ and ER-. Genes associated with the ribosome and oxidative phosphorylation were enriched in the differentially correlated genes between HER2 subtypes. In general, the set of genes identified by DCloc contained more significantly overrepresented terms than the set of genes identified by DCglob.

**Table 3:** Gene enrichment analysis (ER+ vs. ER-)

**Category**	**Catalog**	**DCloc**		**DCglob**	
		***N***	***p***	***N***	***p***
Extracellular matrix	GOTERM_CC_FAT	60	9.6E-13	29	2.8E-01
Cell adhesion	GOTERM_BP_FAT	80	2.0E-07	43	5.5E-01
Cell cycle	GOTERM_BP_FAT	79	2.2E-05	63	3.7E-03
Immune response	GOTERM_BP_FAT	70	6.4E-05		n.s.
Growth factor binding	GOTERM_MF_FAT	22	1.5E-04		n.s.
Organelle fission	GOTERM_BP_FAT	30	1.6E-03	24	4.2E-02
ECM-receptor interaction	KEGG_PATHWAY	18	3.9E-03		n.s.
Ribosome	KEGG_PATHWAY	18	5.5E-03		n.s.
Oxidoreductase	SP_PIR_KEYWORDS	42	8.3E-02	42	2.2E-02

The table shows the most significantly enriched biological themes in the lists of 770 (DCloc) and 630 (DCglob) DC genes. For each functional category, the number of genes in the category (*N*) and the significance of enrichment (Benjamini-Hochberg corrected *p*-value) are shown. Some of the findings of DCglob are not significant (n.s.).

**Table 4:** Gene enrichment analysis (HER2+ vs. HER2-)

**Category**	**Catalog**	**DCloc**		**DCglob**	
		***N***	***p***	***N***	***p***
Translational elongation	GOTERM_BP_FAT	57	1.5E-32	14	3.1E-01
Ribonucleoprotein	SP_PIR_KEYWORDS	83	7.1E-31	46	1.2E-09
Ribosome	KEGG_PATHWAY	55	4.0E-30	14	1.8E-01
Acetylation	SP_PIR_KEYWORDS	303	8.5E-20	276	2.3E-28
Mitotic cell cycle	GOTERM_BP_FAT	77	6.8E-13	65	7.8E-12
Regulation of ubiquitin-protein	GOTERM_BP_FAT	27	3.3E-09	17	6.5E-04
ligase activity during					
mitotic cell cycle					
Immune response	GOTERM_BP_FAT	98	1.0E-08		n.s
Oxidative phosphorylation	KEGG_PATHWAY	28	1.7E-04	30	4.5E-08
Mitochondrion	GOTERM_CC_FAT	93	4.0E-01	106	6.6E-07
Proteasomal ubiquitin-depend-	GOTERM_BP_FAT	27	2.7E-06	20	8.4E-04
ent protein catabolic process					
Mitochondrial membrane part	GOTERM_CC_FAT	26	1.3E-04	25	4.5E-06
MHC protein complex	GOTERM_CC_FAT	13	3.5E-04	7	1.7E-01
Growth factor binding	GOTERM_MF_FAT	22	8.5E-03		n.s
NADH dehydrogenase activity	GOTERM_MF_FAT	10	7.6E-02	11	2.4E-03
ATP synthesis coupled	GOTERM_BP_FAT	12	2.7E-02	12	5.9E-03
electron transport					
Anti-apoptosis	GOTERM_BP_FAT	30	5.0E-02		n.s

The table shows the most significantly enriched biological themes in the lists of 1027 (DCloc) and 804 (DCglob) DC genes. For each functional category, the number of genes in the category (*N*) and the significance of enrichment (Benjamini-Hochberg corrected *p*-value) are shown. Some of the findings of DCglob are not significant (n.s.).

### Heatmap analysis

**Figure 3:**
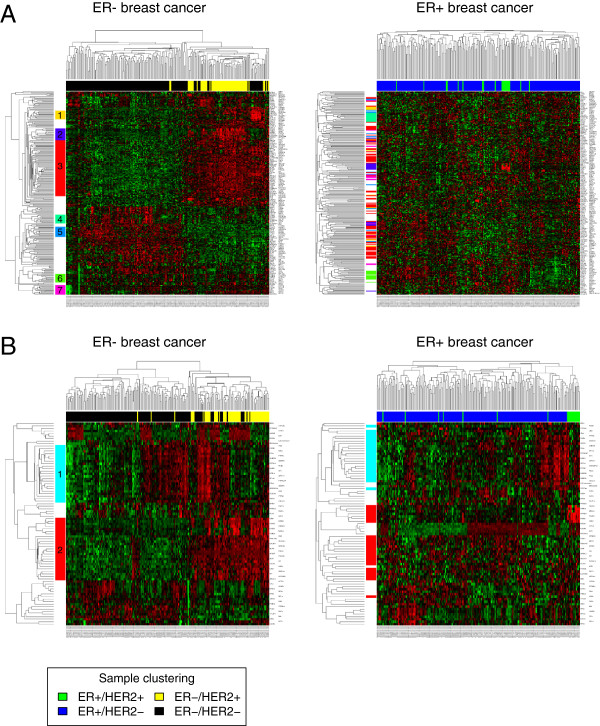
**Heatmaps of genes with higher correlation in ER- tumors compared to ER+ tumors.(A)** Algorithm DCglob: Heatmap of 254 differentially correlated genes (*p*<0.05) in ER- breast cancer (left panel) and in ER+ breast cancer (right panel). Color bars visualize the gene cluster structure in ER- breast cancer and its disorganization in ER+ breast cancer. Seven clusters of genes were identified by cutting the correlation tree at a Pearson correlation of 0.4. **(B)** Algorithm DCloc: Heatmap of 81 differentially correlated genes (*d*>0.3) in ER- breast cancer (left panel) and in ER+ breast cancer (right panel). Color bars as in panel **(A)**.

For each of the subtype comparisons, we generated separate lists of genes that showed a stronger correlation in one of the subtypes compared to the complementary subtype (for example ER- compared to ER+). The resulting four gene lists (ER-, ER+, HER2- and HER2+ subtype) were subjected to hierarchical clustering and heatmap analysis (Figure 3, Additional file 5). The left part of the figures shows a heatmap of the subtype under investigation. Clusters of genes with Pearson correlation greater than 0.4 are marked by colored bars. The right part of the figures shows a heatmap of the complementary subtype. The rearrangement of the colored bars shows the change of the correlation pattern between the subtype under investigation and the complementary subtype.

We analyzed the correlation structure of the differentially correlated genes that showed higher correlation in ER- breast cancer compared to ER+ breast cancer in more detail (Figure 3). Within the differentially correlated genes detected by DCglob, seven clusters (colored bars) were identified by cutting the clustering tree at a Pearson correlation of 0.4. There was a significant overlap between the genes in clusters and marker genes of breast cancer subtypes that were described before, see Table 5.

**Table 5:** Marker gene enrichment analysis

**Cluster**	**Marker gene**	**Marker gene**	***#C***	***#M***	***#*(*C*∩*M*)**	***p***
	**description**	**reference**				
1	IAC	Celis et al. [37]	11	32	5	3.3E-11
2	HER2+	Staaf et al. [39]	12	15	4	6.2E-10
3	AR responsive	Doane et al. [40]	47	70	19	5.2E-32
5	FOXC1 subtype	Ray et al. [41]	13	17	3	5.6E-07

Analysis of the genes identified by DCglob that showed higher correlation in ER- compared to ER+ breast cancer. The gene clusters (*C*) were compared to lists of marker genes (*M*) from the literature. Let #*C* denote the number of genes in the clusters, #*M* the number of marker genes and #(*C* ∩*M*) the number of genes in the intersection. The significance of enrichment was assessed using Fisher’s exact test. *IAC* invasive apocrine carcinomas, *AR* androgen receptor.

Cluster 1 (orange) included ACSM1 and PGDH, two genes that were described as markers for invasive apocrine carcinomas (IACs), a subgroup of ER- breast cancer recently studied by Celis et al. [37,38]. It was enriched with other markers of IAC (Table 5). Cluster 2 (dark-blue) consisted of genes that are up-regulated in HER2+ breast cancer, including ERBB2, GRB7, STARD3 and PSMD3 that are located in the HER2 amplicon [39]. It was significantly enriched for genes of the HER2 amplicon (Table 5). Cluster 3 (red) was highly enriched with marker genes for the androgen-responsive subgroup of ER- breast cancer described by Doane et al. [40]. Cluster 4 (blue-green) contained some cell cycle genes (CDC16, TFDP1). Cluster 5 (light-blue) contained FOXC1, a gene with regulatory and prognostic relevance in triple-negative breast cancer [41]. Cluster 6 (green) contained many genes that are related to ATPase activity (ATP5J, DHX15, CCT8, PSMC6 and ATP5O). Finally, Cluster 7 (purple) contained genes coding for keratins (KRT18, KRT19, KRT7, KRT8), claudins (CLDN3, CLDN4) and CD24. While part of the correlations in Cluster 2 (HER2), Cluster 4 and Cluster 6 (ATPase activity) are preserved in ER+ breast cancer, Cluster 1 (IAC markers), Cluster 3 (AR signaling) and Cluster 5 are completely rearranged in ER+ breast cancer.

DCloc identified only two different clusters. Among others, Cluster 1 (light-blue) contains genes related to the immune response (PTPRC, SIT1, CXCL13, FAIM3, IFI35). Like the third cluster identified by DCglob, the second cluster includes AR and FOXA1. Among the 25 genes present in this cluster, 18 are also present in Cluster 3 of the DCglob analysis. The two clusters identified by DCloc are not completely disarranged in the ER+ subtype, but the correlation between the genes in the clusters is much weaker in the ER+ compared to the ER- subtype.

### Network analysis

**Figure 4:**
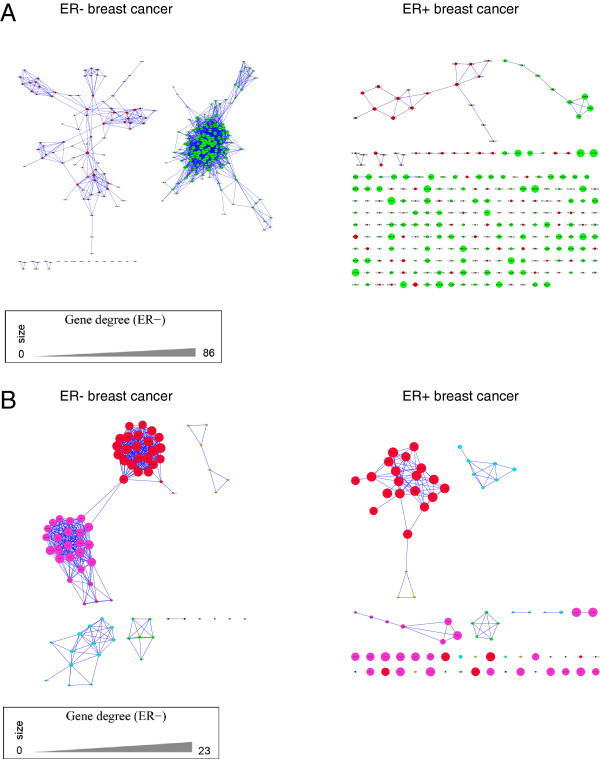
**Correlation networks of genes with higher correlation in ER- tumors compared to ER+ tumors.(A)** Algorithm DCglob, *p*<0.05: Correlation networks in ER- breast cancer (left panel) and ER+ breast cancer (right panel). In this case, almost all the subnetworks present in ER- breast cancer are disorganized in ER+ breast cancer. Genes are connected by an edge if their Pearson correlation is larger than 0.5. The size of nodes in both networks is proportional to the degree of nodes in the network of ER- breast cancer. **(B)** Algorithm DCloc, *d*>0.3: Correlation networks in ER- breast cancer (left panel) and ER+ breast cancer (right panel).

Networks were constructed for the correlation threshold 0.5 (Figure 4, Additional file 5). There are two major connected components identified by DCglob comparing ER- and ER+ breast cancer. The structure of the genes in these clusters is fundamentally different between the ER subtypes. There were also two clusters identified by DCloc. Although the overall structure is less disorganized between the subtypes, the number of edges in the ER- network is higher than those in the ER+ network.

### Reproducibility analysis

**Figure 5:**
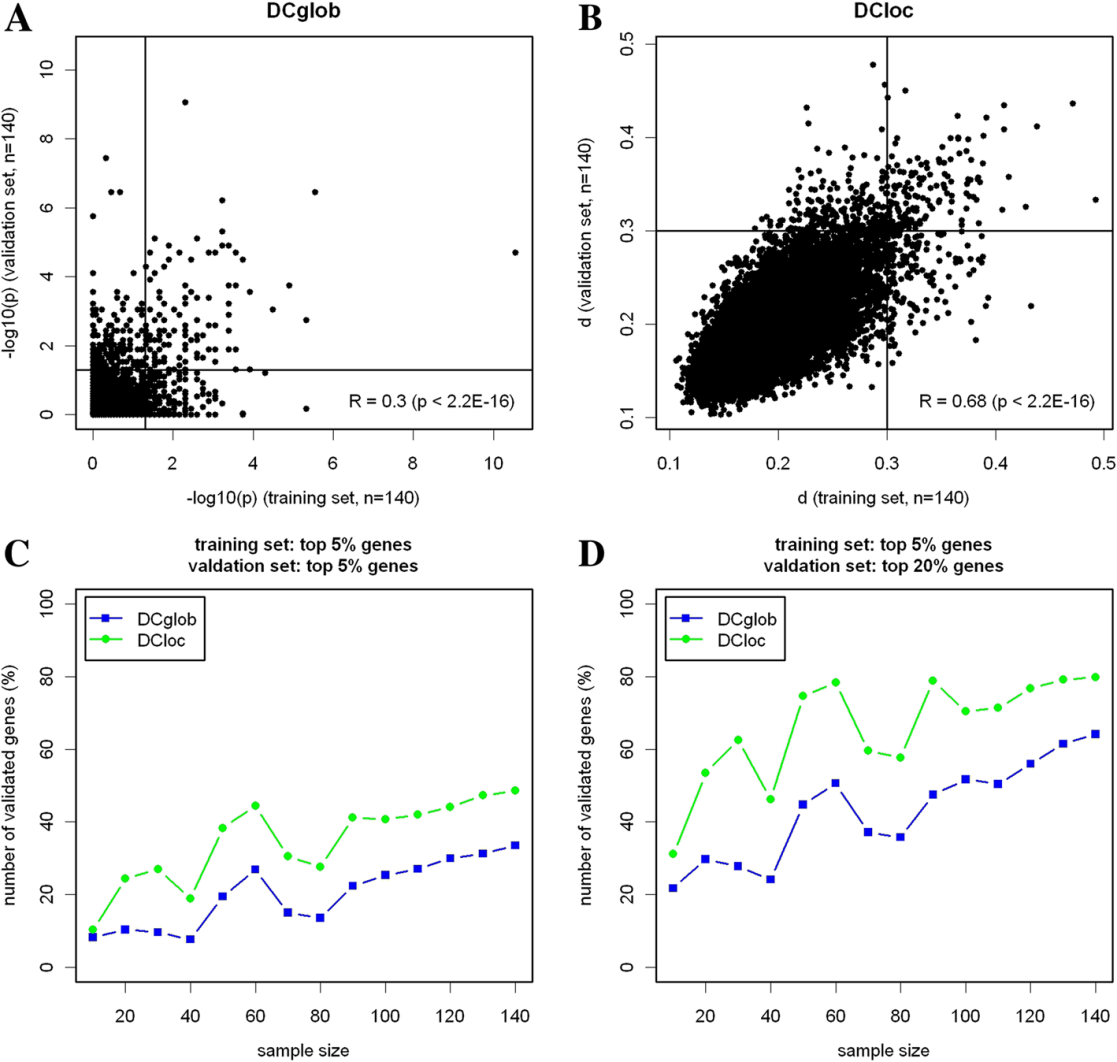
**Analysis of reproducibility of the detections of the topological algorithms.** The reproducibility of the DC analysis of ER- and ER+ breast cancer subtypes is analyzed in two-fold cross-validation. **(A)** Reproducibility of the score *p* (DCglob) calculated in a training set (*n*=140) and in an independent validation set (*n*=140). Lines mark the threshold (*p*=0.05) for the detection of differential correlation. **(B)** Reproducibility of the score *d* (DCloc) calculated in a training set (*n*=140) and in an independent validation set (*n*=140). Lines mark the threshold (*d*=0.3) for the detection of differential correlation. **(C)** Reproducibility analysis of the lists of differentially correlated genes. A series of training and independent validation sets of equal size is randomly drawn from the 1035 ER+ and the 282 ER- patients. A gene detected among the top 5% differentially correlated genes in the training set is considered as validated, if it is among the top 5% DC genes in the validation set. **(D)** As C, but a gene is considered as validated, if it is among the top 20% differentially correlated genes in the validation set.

In a two-fold cross-validation approach, we analyzed the reproducibility of the detections of the topological algorithms. To this end, we randomly drew 10,20,…,140 ER- tumors and 10,20,…,140 ER+ tumors from the study cohort to form training data sets. Then, we randomly drew independent validation data sets of the same sizes. Figure 5A shows a moderate, but highly significant correlation of the statistics *p* (DCglob) between training and validation data sets of 140 samples (Spearman R = 0.30). Figure 5B shows a strong and highly significant correlation of the statistics *d* (DCloc) between the same data sets (Spearman R = 0.68).

Further, we investigated the reproducibility of lists of differentially correlated genes in dependence of the sample size. When comparing the list of the top 5% genes in the training data set to the list of the top 5% genes in the validation data set, the reproducibility raised up to 33% for DCglob and to 49% for DCloc (Figure 5C). When relaxing the reproducibility condition to the list of the top 20% genes in the validation set, this percentage reached 64% for DCglob and 80% for DCloc (Figure 5D).

## Discussion and conclusions

We developed two novel algorithms for the detection of differential correlation (DC) in high-dimensional molecular data. Both approaches are untargeted in the sense that they do not depend on predefined gene modules and start with mapping of the correlation structures to networks. DCglob analyzes global changes of network topology, while DCloc analyzes local changes of the network topology. An innovative ingredient of the algorithms is the analysis of a series of networks that are constructed from a series of correlation thresholds. Therefore, detection of different kinds of correlation changes (strong changes of a few genes or moderate changes of many genes) is feasible. As default setting, the networks are constructed from an equidistant (after Fisher-transformation) series of 100 correlation thresholds between 0 and the maximal correlation in the data set. For the global algorithm, we increased the number of thresholds to 200 to achieve a more precise ranking of the genes. Using 200 instead of 100 thresholds did not significantly change the lists of DC genes. For both algorithms, a statistic for the strength of DC (*p* or *d*) is calculated as average of topological changes over all correlation thresholds. Using averaging as method for summary ensures that changes at different correlation thresholds are taken into account with equal weight. This i appropriate in a situation without prior knowledge on the strength and biological relevance of correlation changes.

An essential part of the analysis is to work with two equal-sized sample groups that are compared for differential correlations. The number of available HER2+ samples was 208 and therefore we randomly drew 208 ER+, 208 ER- and 208 HER2- samples for analysis. Unequal samples size would lead to a different sensitivity and/or specificity for the detection of correlations and therefore would lead to false positive discoveries in the DC analysis. We therefore recommend to work with equal-sized sample groups.

The results of DCloc and DCglob were consistent: Pearson correlations between −*p* and *d* were strong and highly significant (*p*<2E-16) for the analysis of estrogen receptor status (R = 0.59) and the analysis of HER2 status (R = 0.64). Extensive subsampling analysis was carried out to demonstrate the significance of the findings, and to estimate FDRs associated with the lists of differentially correlated genes. Both algorithms detected a significant number of differentially correlated genes in molecular subtypes of breast cancer compared with the null model. In the analysis of breast cancer subtypes, DCloc turned out to be more sensitive than DCglob (Figure 2C). This was in line with a higher number of significantly enriched functional categories for DCloc(Table 3). Additionally, the performance of DCloc was considerable better than DCglob in terms of reproducibility when analyzing two independent breast cancer data sets (Figure 5). Assuming that 1/3 of the detected genes should reproduce in two-fold cross-validation, 90 patients in each group were appropriate for DCloc and 130 patients in each group were appropriate for DCglob. However, the results of the global algorithm were more easily interpretable in the heatmap analysis (Figure 3).

Gene enrichment analysis revealed significantly enriched terms within the lists of differentially correlated genes. We tested heatmaps and networks as tools for visualization and in-depth analysis of the correlation changes. Clustering and heatmap analysis turned out to be particularly useful for a biological interpretation of the correlation patterns. ER- tumors tend to be more aggressive and are more difficult to treat than ER+ tumors. Therefore, we analyzed the correlation patterns in ER- tumors in more detail. Interestingly, among the clusters of genes detected by DCglob, there were many genes that could serve markers for substratification of the ER- or the triple-negative subtype (see Table 5).

We detected different types of changes in the correlation patterns between ER+ and ER- breast cancer. For example, the structure of the cluster associated with the HER2 amplicon (Cluster 2) was relatively well preserved. However, there was a difference in correlation strength, which can be possibly explained by the unequal distribution of the HER2 tumors between the ER+ and the ER- subtype (see Table 1). In contrast, there were gene clusters with strong correlation in ER- breast cancer and almost no correlation in ER+ breast cancer (Clusters 1, 3 and 5).

As an example, the genes in Cluster 1 were strongly correlated in ER- breast cancer, but most of them were uncorrelated in ER+ breast cancer. In the marker gene enrichment analysis, we found a highly significant overlap between these genes and marker genes of invasive apocrine carcinomas (IACs). In fact, the genes of Cluster 1 were highly expressed in a small subgroup of triple-negative (ER- and HER2-) breast cancer (10 tumors), but poorly expressed in the remaining ER- tumors and in all ER+ tumors. The two major marker genes for IACs [37], PGDH and ACSM1, were highly differentially correlated, but not differentially expressed between the ER- and the ER+ subtype.

Interestingly, the genes in Cluster 3 were highly expressed in the ER-/HER2+ tumors and the IACs, but not in the remaining triple-negative tumors. The marker gene enrichment analysis demonstrated a highly significant overlap of this cluster with AR signaling. The AR gene itself turned out to be considerably higher expressed in ER+ tumors compared to ER- tumors (fold change =3.01, *p*=1.2E-30). This result is in agreement with immunohistochemical data showing that the number of androgen receptor positive tumors is larger in the ER+ subtype [42]. Further analysis showed that the expression of most of the genes of the AR signaling cluster was high in the ER+ tumors, but variable in the ER- tumors. Thus, the low correlation of the AR signaling genes in ER+ tumors is a consequence of the missing variance of the pathway in these tumors, where it is always highly expressed. These observations suggest that AR signaling is always active in ER+ tumors, while it is under regulation (active or inactive) in ER- tumors. AR signaling based stratification is of interest in the ER- subtype, but not in the ER+ subtype, in line with a recent result that high AR protein expression was associated with better survival in triple-negative breast cancer [42].

In summary, functionally relevant pathways (Table 3, 4 and 5) could be identified that show highly correlated gene expression in one of the subtypes, but not in the complementary subtype. Co-expression of a pathway is likely to be a consequence of pathway regulation, for example by transcription factors. A pathway highly expressed in a cancer subtype is potentially supporting or even essential for the growth of tumors cells in this kind of tumor. Thus, the clusters identified by DC analysis can be beneficial for patient stratification and can represent interesting targets for new therapies. In this context we re-identified the HER2-amplicon that is successfully targeted by trastuzumab or other anti-HER2 drugs [43].

These examples illustrate that DC analysis, in particular network topology based approaches, can help to identify biologically important gene clusters beyond the results of conventional DE analysis. Using DCloc and DCglob, we detected hundreds of differentially correlated genes in the molecular subtypes of breast cancer, while keeping the FDR under control. In a two-fold cross-validation approach we showed that results of both algorithms were reproducible in an independent data set.

Within the last decade, a multitude of methods and algorithms were developed for the detection of differential correlations. The algorithms address different biological questions and it is difficult to decide which of the algorithms works best in terms of power and of biological interpretability. However, statistical properties like specificity and reproducibility could be evaluated in a comparable way for many of the algorithms. We believe that statistical evaluation should be stronger emphasized in future studies of differential correlations.

## Abbreviations

DC: Differential correlation; DE: Differential expression; FDR: False discovery rate; ER: Estrogen receptor; HER2: Human epidermal growth factor receptor 2; IHC: Immunohistochemistry; BH: Benjamini-Hochberg; IAC: Invasive apocrine carcinoma; AR: Androgen receptor.

## Competing interests

The authors declare that they have no competing interests.

## Authors’ contributions

JB conceived the study. MB and JB designed the algorithms. MB implemented the algorithms and analyzed data. All authors contributed to biological analysis and interpretation of the results. MB and JB wrote the manuscript. All authors read and approved the final version of the manuscript.

## Supplementary Material

Additional file 1 **R code for calculation of differential correlations.** There are two functions that run independently of each other: DCglob compares the global topology of correlation networks, while DCloc compares the local topology of the correlation networks. Click here for file

Additional file 2 **Subtype classification by ER and HER2 expression.** (A) Expression level of ER (205225_at) in 1317 breast cancer samples. A tumor was classified ER+ whenever the ER expression level was larger than 10.(B) Expression level of HER2 (216836_s_at). A tumor was classified HER2+ whenever the expression level of HER2 was larger than 12. Click here for file

Additional file 3 **Differential correlation in ER+ and ER- breast cancer.** Table of the genes represented by the Affymetrix HG-U133A microarray with the strength of differential correlation. All the results were obtained comparing a set of 208 ER+ and 208 ER- tumors. For DCloc, the sum of topological dissimilarities, the sum of the absolute values of topological dissimilarities (*d*) and the mean number of neighbors in the ER+ and the ER- network are shown. For DCglob, the stability of differential correlation (*p*) and the subtype where the gene shows higher correlation are shown. Finally, the table contains information on differential expression (DE) between ER+ and ER- subtype. Click here for file

Additional file 4 **Differential correlation in HER2+ and HER2- breast cancer.** Table analogous to Additional File 2 for the comparison of 208 HER2+ breast cancer samples with 208 HER2- breast cancer samples. Click here for file

Additional file 5 **Heatmaps and networks of genes with high correlation in ER+, HER2- and HER2+ tumors.** Heatmaps and networks analogous to those shown in Figure 3 and Figure 4 for the differentially correlated genes that showed high correlation in ER+ tumors (p. 1-2), HER2- tumors (p. 3-4), and HER2+ tumors (p. 5-6). Click here for file
