# Supplementary figures and images for: New network topology approaches reveal differential correlation patterns in breast cancer
Source: BMC Syst Biol. 2013 Aug 15;7:78. doi: 10.1186/1752-0509-7-78 (PMC3848818; doi:10.1186/1752-0509-7-78)

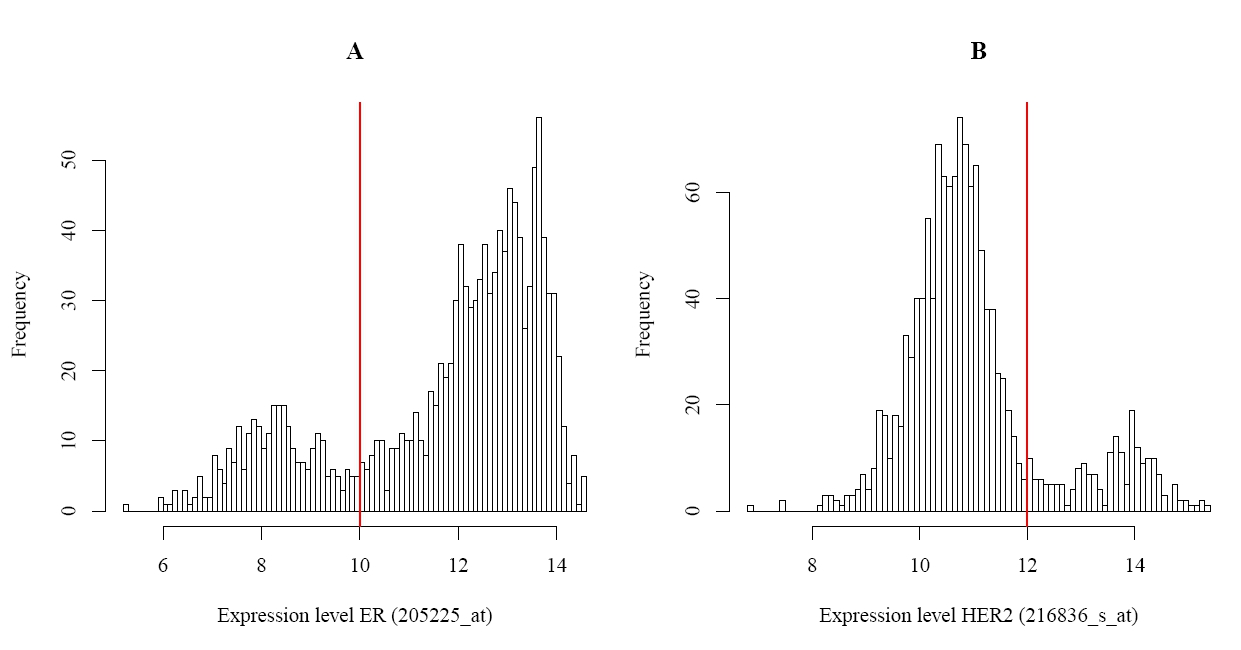

Supplement: Additional file 2 — Subtype classification by ER and HER2 expression. (A) Expression level of ER (205225_at) in 1317 breast cancer samples. A tumor was classified ER+ whenever the ER expression level was larger than 10.(B) Expression level of HER2 (216836_s_at). A tumor was classified HER2+ whenever the expression level of HER2 was larger than 12. [file 1752-0509-7-78-S2.jpeg]
