# Supplementary material for: New network topology approaches reveal differential correlation patterns in breast cancer
Source: BMC Syst Biol. 2013 Aug 15;7:78. doi: 10.1186/1752-0509-7-78 (PMC3848818; doi:10.1186/1752-0509-7-78)
Supplement: Additional file 5 — Heatmaps and networks of genes with high correlation in ER+, HER2- and HER2+ tumors. Heatmaps and networks analogous to those shown in Figure 3 and Figure 4 for the differentially correlated genes that showed high correlation in ER+ tumors (p. 1-2), HER2- tumors (p. 3-4), and HER2+ tumors (p. 5-6). [file 1752-0509-7-78-S5.pdf]

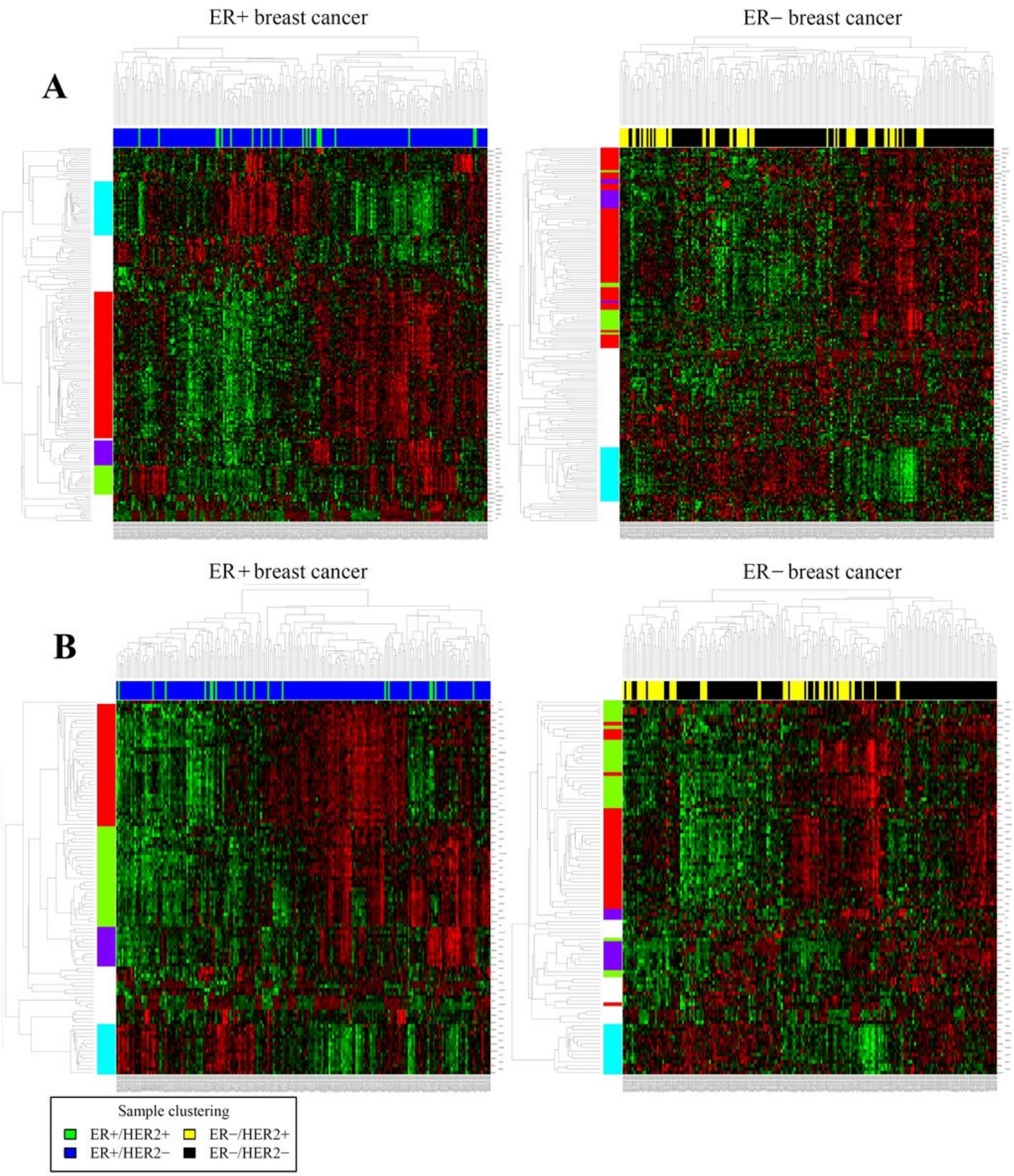

Heatmaps of genes with higher correlation in ER+ tumors compared to ER- tumors. (A) Algorithm `DCglob`: Heatmaps of 166 differentially correlated genes ($p < 0.05$) in ER+ breast cancer (left panel) and in ER- breast cancer (right panel). Color bars visualize the gene cluster structure in ER+ breast cancer and the corresponding structure in ER- breast cancer. They were identified by cutting the correlation tree at a Pearson correlation of 0.4. (B) Algorithm `DCloc`: Heatmaps of 104 differentially correlated genes ($d > 0.3$) in ER+ breast cancer (left panel) and in ER- breast cancer (right panel). Color bars as in panel (A).

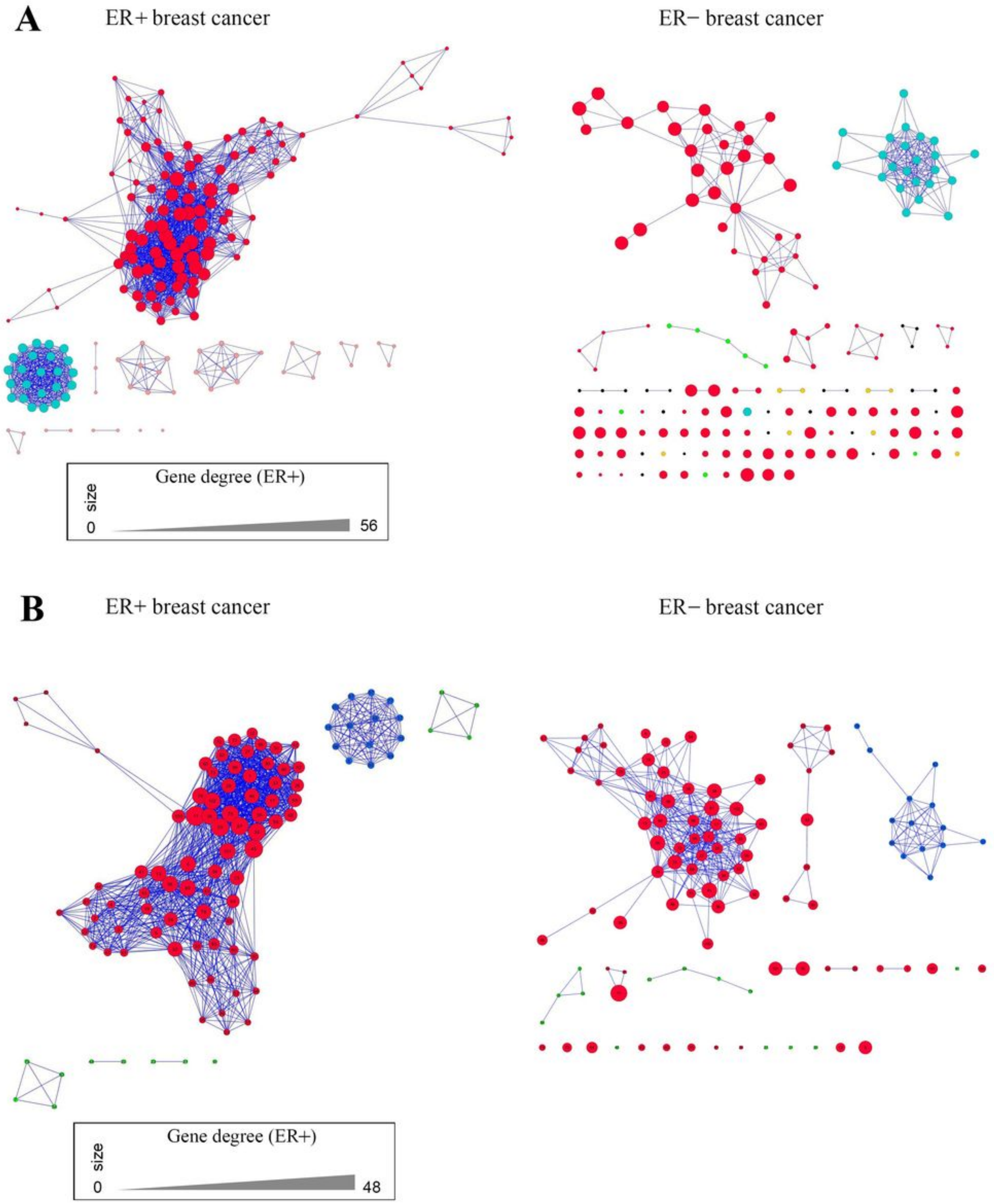

Correlation networks of genes with higher correlation in ER+ tumors compared to ER- tumors. (A) Algorithm `DCglob`, $p < 0.05$: Correlation networks in ER+ breast cancer (left panel) and ER- breast cancer (right panel). Genes are connected by an edge if their Pearson correlation is larger than 0.5. The size of nodes in both networks is proportional to the degree of nodes in the network of ER+ breast cancer. (B) Algorithm `DCloc`, $d > 0.3$: Correlation networks in ER+ breast cancer (left panel) and ER- breast cancer (right panel).

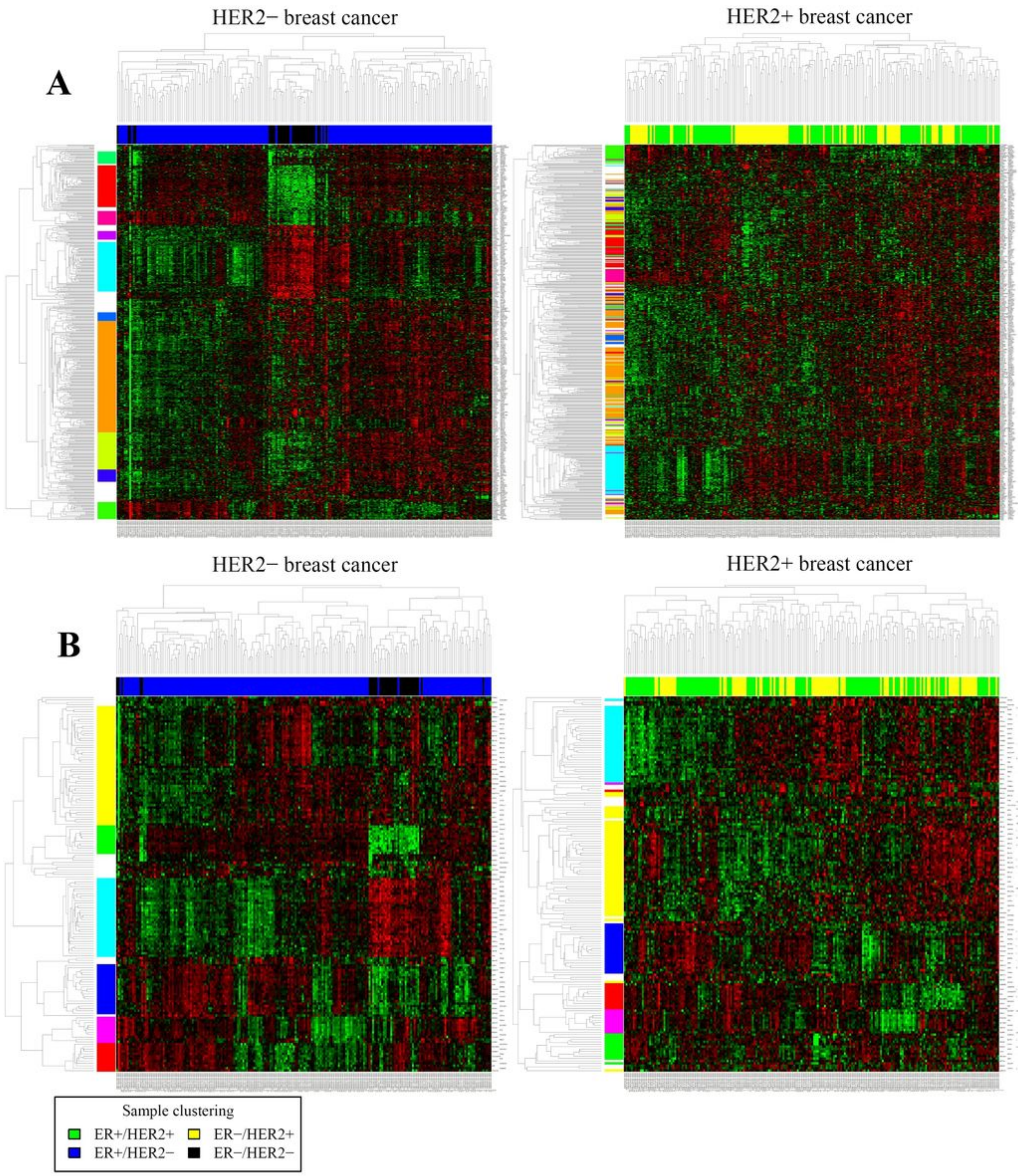

Heatmaps of genes with higher correlation in HER2- tumors compared to HER2+ tumors. (A) Algorithm `DCglob`: Heatmaps of 485 differentially correlated genes ($p < 0.05$) in HER2- breast cancer (left panel) and in HER2+ breast cancer (right panel). Color bars visualize the gene cluster structure in HER2- breast cancer and its disorganization in HER2+ breast cancer. They were identified by cutting the correlation tree at a Pearson correlation of 0.4. (B) Algorithm `DCloc`: Heatmaps of 157 differentially correlated genes ($d > 0.3$) in HER2- breast cancer (left panel) and in HER2+ breast cancer (right panel). Color bars as in panel (A).

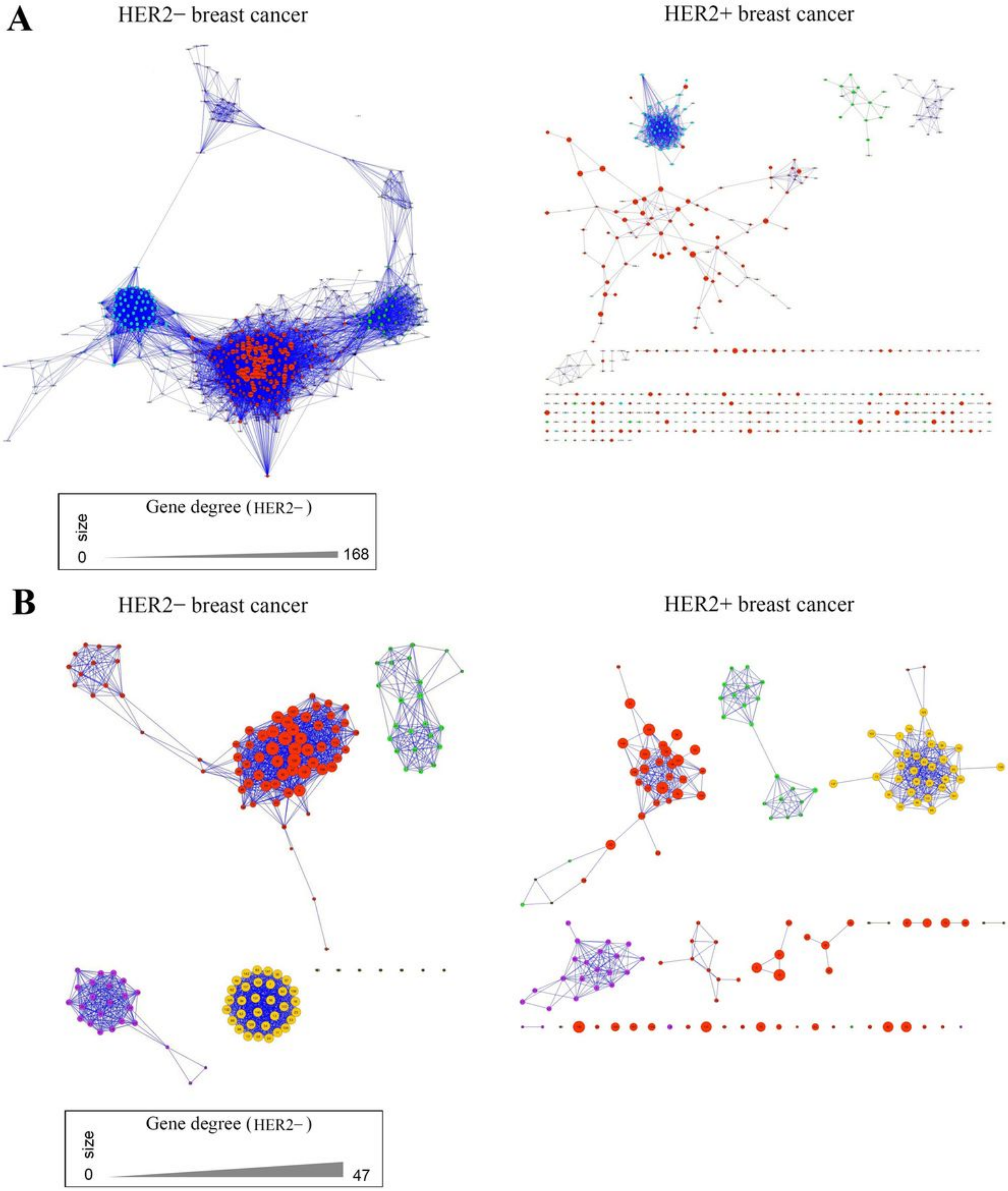

Correlation networks of genes with higher correlation in HER2- tumors compared to HER2+ tumors. (A) Algorithm `DCglob`, $p < 0.05$: Correlation networks in HER2- breast cancer (left panel) and HER2+ breast cancer (right panel). Genes are connected by an edge if their Pearson correlation is larger than 0.5. The size of nodes in both networks is proportional to the degree of nodes in the network of HER2- breast cancer. (B) Algorithm `DCloc`, $d > 0.3$: Correlation networks in HER2- breast cancer (left panel) and HER2+ breast cancer (right panel).

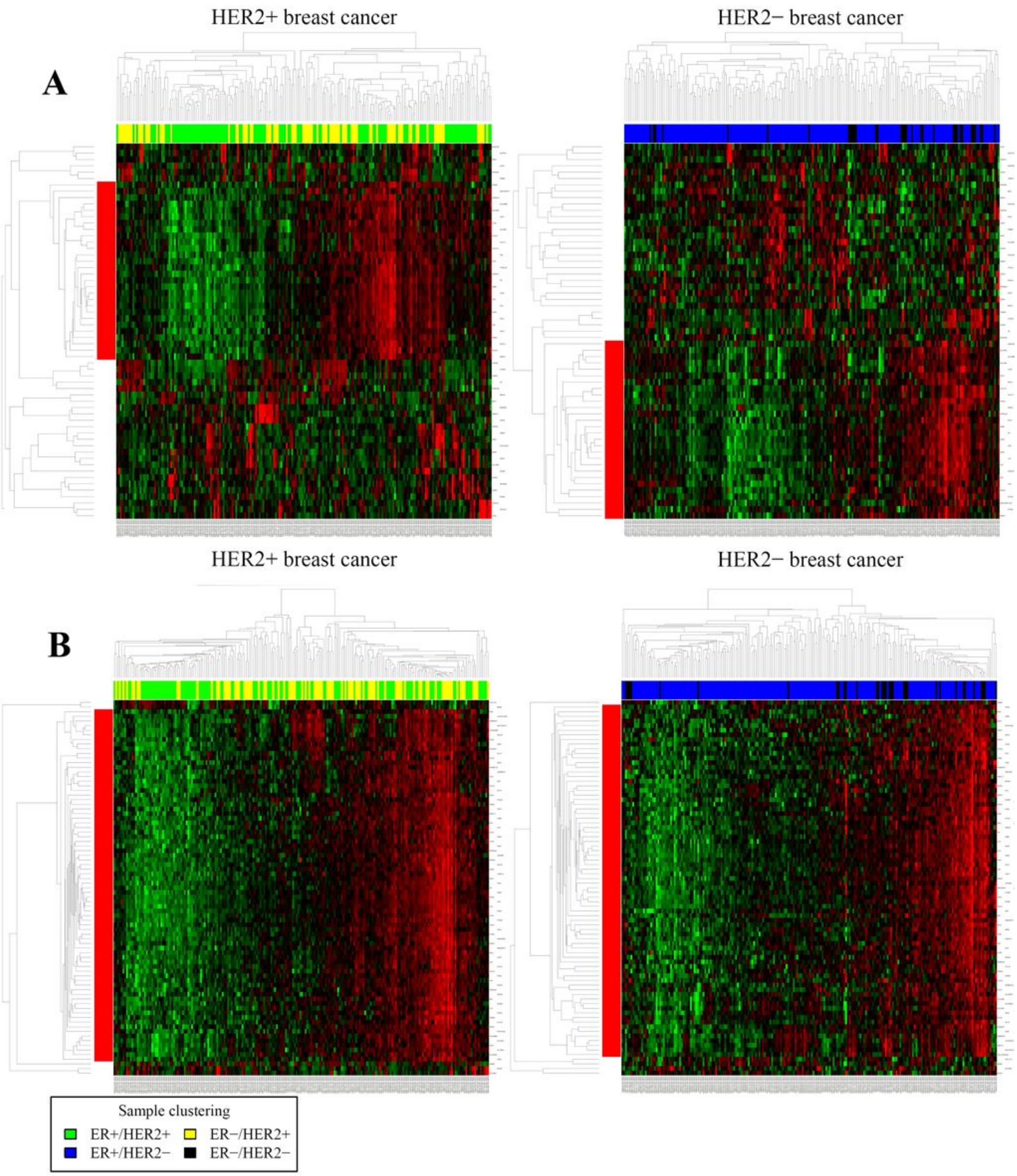

Heatmaps of genes with higher correlation in HER2+ tumors compared to HER2- tumors. (A) Algorithm `DCglob`: Heatmaps of 59 differentially correlated genes ($p < 0.05$) in HER2+ breast cancer (left panel) and in HER2- breast cancer (right panel). Color bars visualize the gene cluster structure in HER2+ breast cancer and the structure in HER2- breast cancer. They were identified by cutting the correlation tree at a Pearson correlation of 0.4. (B) Algorithm `DCloc`: Heatmaps of 81 differentially correlated genes ($d > 0.3$) in HER2+ breast cancer (left panel) and in HER2- breast cancer (right panel). Color bars as in panel (A).

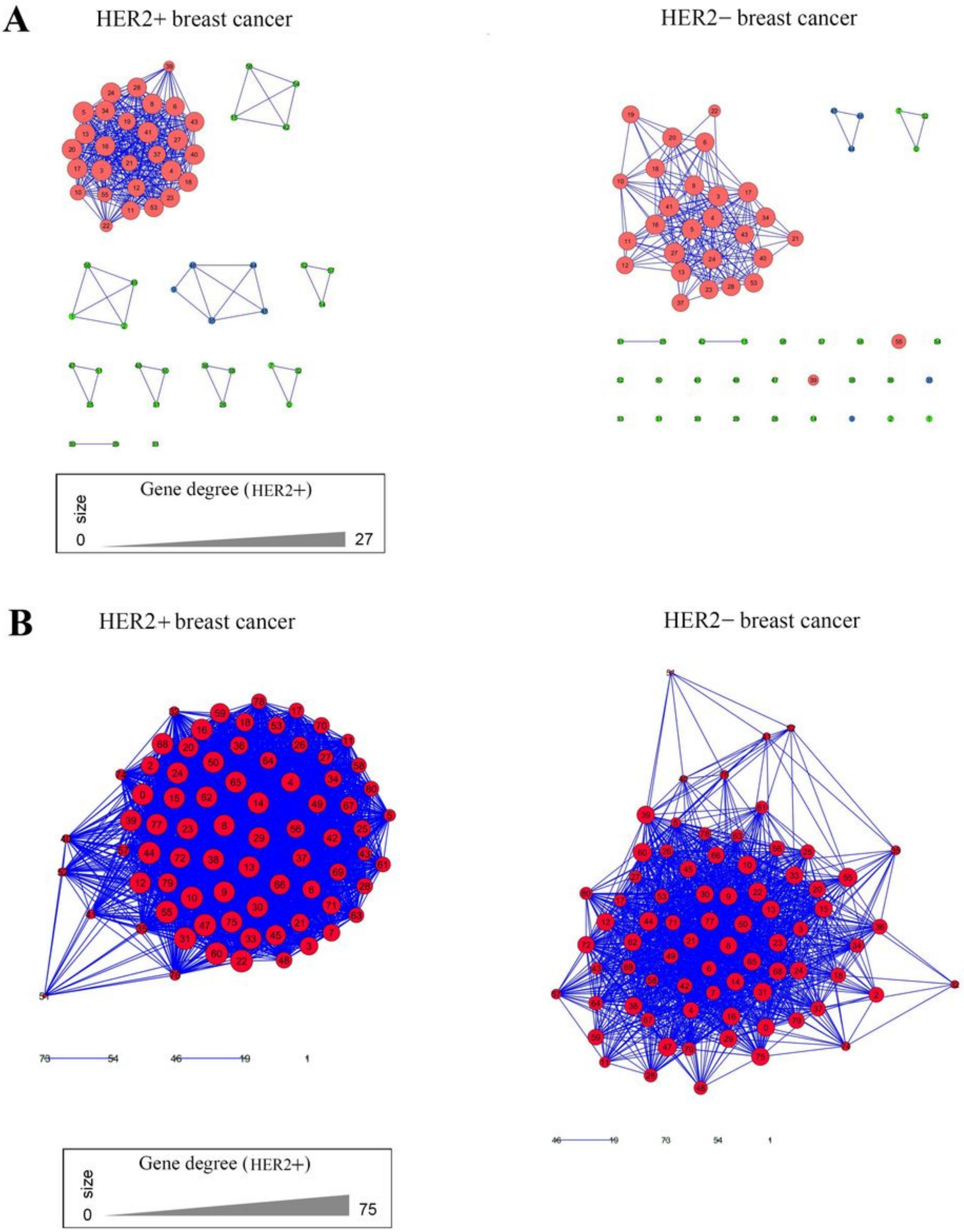

Correlation networks of genes with higher correlation in HER2+ tumors compared to HER2- tumors. (A) Algorithm `DCglob`, $p < 0.05$: Correlation networks in HER2+ breast cancer (left panel) and HER2- breast cancer (right panel). Genes are connected by an edge if their Pearson correlation is larger than 0.5. The size of nodes in both networks is proportional to the degree of nodes in the network of HER2+ breast cancer. (B) Algorithm `DCloc`, $d > 0.3$: Correlation networks in HER2+ breast cancer (left panel) and HER2- breast cancer (right panel).
